# Zn-Co metal organic frameworks coated with chitosand and Au nanoparticles for chemo-photothermal-targeted combination therapy of liver cancer

**DOI:** 10.3389/fonc.2023.1110909

**Published:** 2023-04-19

**Authors:** Congling Yang, Santosh K. Tiwari, Lianshan Guo, Guanghui An, Heming Zheng, JianFeng Huang, Li Jiang, Zhihao Bai, Yanqiu Zhu, Nannan Wang

**Affiliations:** ^1^ Key Laboratory of New Processing Technology for Nonferrous Metals and Materials, Guangxi Institute Fullerene Technology (GIFT), Ministry of Education, School of Resources, Environment and Materials, Guangxi University, Nanning, Guangxi, China; ^2^ Faculty of Chemistry, University of Warsaw, Warsaw, Poland; ^3^ Department of Chemistry, NMAM Institute of Technology, Nitte (Deemed to be University) Nitte, Karnataka, India; ^4^ Department of Emergency, The Second Affiliated Hospital of Guangxi Medical University, Nanning, China; ^5^ Department of Nephrology, The Second Affiliated Hospital of Guangxi Medical University, Nanning, China; ^6^ Department of Radiation Oncology, The First Affiliated Hospital of Guangxi Medical University, Nanning, China; ^7^ College of Chemistry and Chemical Engineering, Guangxi University, Nanning, China

**Keywords:** alleviating hypoxia, Zn-Co MoF, chemo-photothermal therapy, pH sensitivity, drug delivery

## Abstract

The toxic effects of chemotherapy drugs on normal tissues are still a major limiting factor in cancer treatment. In this paper, we report a metal-organic framework (Zn-Co ZIF) with chitosan-coated outer layer as a carrier for the drug adriamycin hydrochloride (DOX), a treatment for liver cancer, as a novel anti-cancer nanodrug-enhanced carrier. Gold nanoparticles, a good photothermal conversion agent, were combined with the target SH-RGD during surface functionalisation to prepare Zn-Co ZIF@DOX-CS-Au-RGD (ZD-CAR), a nanoplatform with good photothermal conversion properties and targeting for combined liver cancer therapy. ZD-CAR was developed after RGD accurately targeted the tumour and entered the tumour microenvironment (TME), it cleaves and releases the liver cancer therapeutic agent (DOX) in a weak acidic environment to effectively kill tumour cells. The metal skeleton cleavage releases Co^2+^, which catalyzes the production of oxygen from H_2_O_2_ to alleviate the tumour hypoxic environment. The dissolved oxygen could reach 14 mg/L after adding 80 mg/mL of ZD-CAR. Meanwhile, gold nanoparticles could convert light energy into heat energy under 808 NIR irradiation to induce local superheating and kill tumour cells. In summary, this study developed a nanoplatform that combines chemo-photothermal-targeted therapy. It has shown good therapeutic effeciency in cellular experiments and performance tests and has promising applications in anti-cancer therapy.

## 1 Introduction

Although medical technology has made great progress, cancer remains one of the leading causes of human mortality (1–6). Traditional treatments, such as surgery and chemotherapy have discouraged many people because surgery is expensive, chemotherapy drugs are usually not sufficient to kill cancer cells, the drugs have short circulation time in the body, and a large portion of the drugs is absorbed by normal tissues before they reach the tumor site. Thus, their damage to normal tissues is hard to ignore (7–9). Therefore, several attempts have been made to research a new drug delivery method in recent years, which can achieve high therapeutic efficacy with higher rates of drug loading. The method needs to accurately release drugs upon reaching the target site, must have good biocompatibility and should be environmental friendly (10–12).

So far, metal-organic frameworks (MOFs) which consist of metal ions and organic ligands are frequently prescribed by many biomedical researchers as promising nanocarriers due to their adjustable size, very high specific surface area inside and outside their pores, better biocompatibility, and good pH responsiveness (13–16). Here, we chose Zn-Co ZIF (50% Co) as the carrier for the nanocarrier platform, which is a member of the vast MOFs system. Zn-Co ZIF nanoparticles were prepared using previously known methods, and it is understood from previous literatures that a ratio of around 50% Zn-Co ions has the most suitable size and properties (17–20). Thus, ZIF with 50% Zn-Co ratio was chosen as a carrier for DOX and it was modified with chitosan in order to make it biocompatible (21–23). In the weakly acidic tumor microenvironment, the metal skeleton would cleavage and release DOX (24–27), thus allowing it to act precisely at the tumor site. This effectively reduces the toxic effects of DOX on normal tissue during its transport.

Liver cancer is one of the most common types of cancers. DOX (Adriamycin) has shown good efficacy in the treatment of liver cancer, but it has poor therapeutic activity and side effects on normal tissues, such as hepatotoxicity (28). These limit its clinical applications. The use of a metal-organic skeleton to deliver it to the tumor site can reduce its risk to normal tissue to some extent (29).

Compared to single treatment modalities, the combination of chemo-photothermal therapy (30, 31) is now considered as a treatment with considerable results. Photothermal therapy (PTT) is popular as a simpler and more protective treatment for normal tissues. This is because it can be used to ablate tumors (32) in a variety of ways through photothermal therapy and it can utilize thermal energy to accelerate the release of heat. At the heart of photothermal therapy are photothermal converters, of which precious metal converters (33–35), gold nanoparticles (Au NPs), have good photothermal conversion efficiency (36, 37). At the same time, the hypoxic environment (38–41) within the tumor is an important limiting factor for photothermal power therapy, and improving the tumor’s hypoxic environment in every way is an integral step in photothermal therapy.

In general, stimulatory drug delivery systems (42, 43) requiring the combination of chemotherapy and photothermal therapy require well-designed nano-delivery platforms to achieve synergistic therapies. But, the nano-delivery platforms also have many drawbacks, such as low drug delivery rates, premature drug leakage by cleavage before reaching the target area, and poor biodegradability of the materials themselves, which are the focus of this work.

**Scheme 1:**
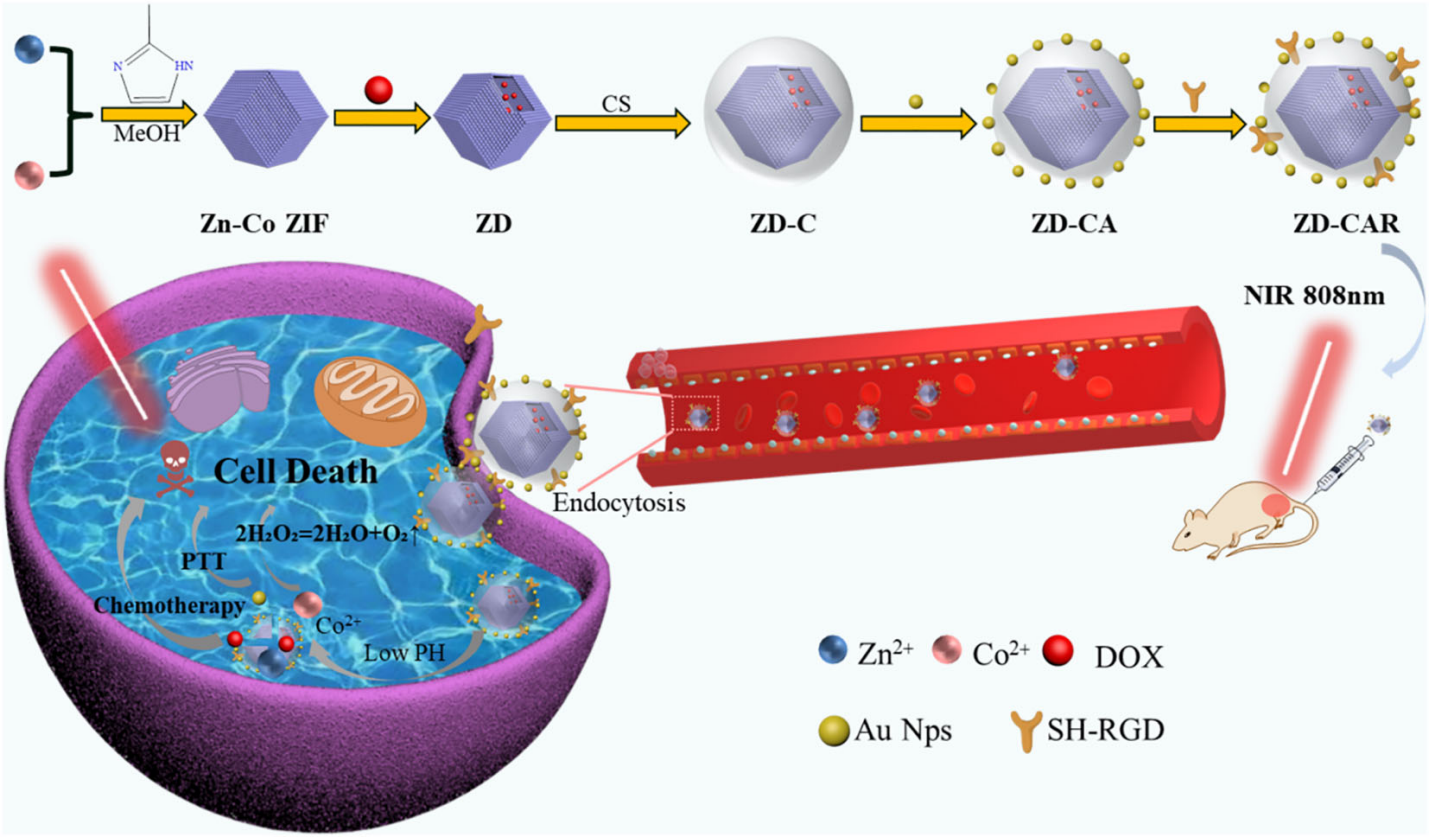
Synthesis process of the ZD-CAR nano-drug delivery platform and its schematic representation of the synergistic effect of chemotherapy and photothermal therapy and relief of hypoxia within tumor cells.

In this research, a novel drug delivery system has been prepared by combining chemotherapy and photothermal therapy for liver cancer, using one of the metal-organic frameworks (MOFs), Zn-Co ZIF. As shown in **Scheme 1**. It has the unique property of loading the chemotherapeutic drug adriamycin hydrochloride (DOX) and releasing the drug. This is done by cleaving in a slightly acidic tumor microenvironment and producing Co^2+^, which can catalyze H_2_O_2_ to produce oxygen. Generation of O_2_ can alleviate the tumor hypoxic environment (31, 38). Meanwhile, for the purpose of improving the biocompatibility, chitosan was covered on the outside of the nano particles. Also, precious metal photo-thermal converters Au NPs were introduced, which could convert light into heat to kill tumor cells under 808 nm NIR irradiation and exhibited good photo stability (34). In order to achieve accurate targeting of tumor cells, mercapturized RGD (SH-RGD) was introduced; it can form strong Au-S bonds with gold nanoparticles, and RGD can accurately target tumor cells and reduce the drug toxicity to normal tissues. *In vitro* experiments have demonstrated that Zn-Co ZIF@DOX-CS-Au-RGD (ZD-CAR) has good anti-tumor efficacy with no significant adverse effects (44–46). Therefore, the currently prepared nano-drug delivery system has good potential as a novel therapeutic platform to combine chemotherapy and photothermal therapy for tumor eradication.

## 2 Materials and methods

### 2.1 Materials

The following materials were used for the study: 2-Methylimidazole (C_4_H_6_N_2_, 2-MIM), Doxorubidn hydrochloride (DOX), chitosan (CS), mercaptopropionic acid (MPA), zinc nitrate hexahydrate (Zn(NO_3_)_2_•6H_2_O), chloroauric acid, cobaltous nitrate hexahydrate (Co(NO_3_)_2_-6H_2_O), methanol, 1-Ethyl-(3-dimethylaminopropyl) carbodiimide hydrochloride (EDC), N-Hydroxysuccinimides (NHS), SH-RGD and sodium borohydride, hydrogen peroxide (H_2_O_2_, 30%). Penicillin/Streptomycin Dual Antibody, Propidium iodide (PI), DMEM, Calcein-AM, DMSO and Fetal calf serum and Trypsin.

### 2.2 Synthesis of Zn-Co ZIF

Zn-Co ZIF was prepared using the method in the literature by adjusting the ratio of zinc nitrate hexahydrate to cobalt nitrate hexahydrate to 1:1. 366 mg of zinc nitrate hexahydrate. 358 mg of Co(NO_3_)_2_·6H_2_O was dissolved in 100 mL of methanol and mixed well. 1.620 g of 2-MIM was dissolved in 100 mL of methanol and mixed well. Then, the mixture of the metal ion was poured into the 2-MIM solution and stirred for 12 h. It was washed with methanol and centrifuged. Then, it was dried to obtain Zn-Co ZIF product (17).

### 2.3 Synthesis of Zn-Co ZIF@DOX nanoparticles

After preparing Zn-Co ZIF according to the literature method, DOX was then encapsulated into the MOF structure using the physical encapsulation method. This was achieved by mixing DOX and Zn-Co ZIF in an ethanol system and stirred for 12 h. The product was centrifuged at 8000 rpm for 10 min and washed with ethanol to obtain the DOX-encapsulated Zn-Co ZIF@DOX (ZD) system. Subsequently, the successful encapsulation of DOX was verified by testing the reduction in BET (Brunner-Emmet-Teller). The load efficiency of DOX can be calculated by the following equation:


(1) $$Loading\;efficiency\left(\%\right)=[1-\frac{{\text{C}}_{1}{\text{V}}_{1}{\text{+C}}_{2}{\text{V}}_{2}}{\text{CV}}]\;\times \;100\%$$

where C_1_/C_2_ is the concentration of the supernatant after the first or second (47) centrifugation, and V_1_/V_2_ is the volume of the supernatant after the first or second centrifugation, C is the concentration before the centrifugation of ZD, and V is the total volume before the centrifugation of ZD. The encapsulation efficiency can be calculated as 53%. The drug loading capacity can be calculated by the following equation:


(2) $$LC=\frac{weight\;of\;initial\;DOX-weight\;of\;DOX\;in\;discrded\;solutions}{weight\;of\;ZD-CAR\;NPs}\times 100\%$$

The calculated drug loading capacity is 15.67% (48–50).

### 2.4 Synthesis of Zn-Co ZIF@DOX-CS

Chitosan is a class of natural polymer material, which can show different molecular structures at different pH levels. Generally, it is polymerized in alkaline conditions. By adjusting the pH, the purpose of wrapping the chitosan outside the material can be achieved. The ZD particles obtained in the previous step were dissolved in aqueous solution. 10 mL of 10% chitosan solution and 1 mL of glutaraldehyde were added to the solution. Then, it was stirred for 6 h. It was washed, centrifuged and dried. Thereafter, ZD-C particles were obtained.

### 2.5 Synthesis of Au NPs

Au NPs were prepared using the literature method and nanoparticles with a diameter of around 40 nm were obtained (51). The obvious nanoparticle morphology can be seen by SEM, and the Au elements can also be seen by their EDS score. This shows that the gold nanoparticles were successfully prepared. The specific preparation method is:First, take 1mL of dilute solution of chloroauric acid (1%), add water to configure 100mL of 0.01% chloroauric acid solution, pour it into a 250mL three-necked flask, put the three-necked flask into an oil bath, adjust the temperature to 130°C for heating, observe the boiling of the chloroauric acid solution and then adjust the temperature to 100°C. After the temperature of the apparatus is stable, add 1% trisodium citrate to the system, turn on the oil bath magnetic stirrer to homogeneous speed, observe that the colour of the solution changed to purplish red when the apparatus was timed to continue heating for 15 min. Finally, the three-necked flask was placed in a room temperature water bath and stirred until the temperature of the gold sol was reduced to room temperature.

### 2.6 Synthesis of Zn-Co ZIF@DOX-CS-Au

The Au nanoparticles were attached to ZIF@DOX-CS by physical linkage. This was achieved by adding 500 μL tetra-arm mercaptopropionic acid and 200 μL 3-(2-Pyridinedimercapto) propionic acid N-hydroxysuccinimide ester (SPDP) and stirred for 3 h. After they were centrifuged, 500 μL of the prepared Au NPs was added to the ZD-C solution. It was stirred smoothly to attach the Au NPs to the outside of the ZD-C. Then, ZD-CA nanoparticles were obtained.

### 2.7 Synthesis of ZD-CAR nanoparticles

The target products ZD-CAR nanoparticles were obtained by attaching mercaptopropionic acid and SH-RGD to the surface of ZD-CA nanoparticles through the action of Au-S covalent bonds. This was done by adding 2 mL RGD (3.4 mg/mL) and 500 μL mercaptopropionic acid. It was stirred for 5 h and centrifuged. Then, ZD-CAR was obtained.

### 2.8 Characterization

**Figure 1:**
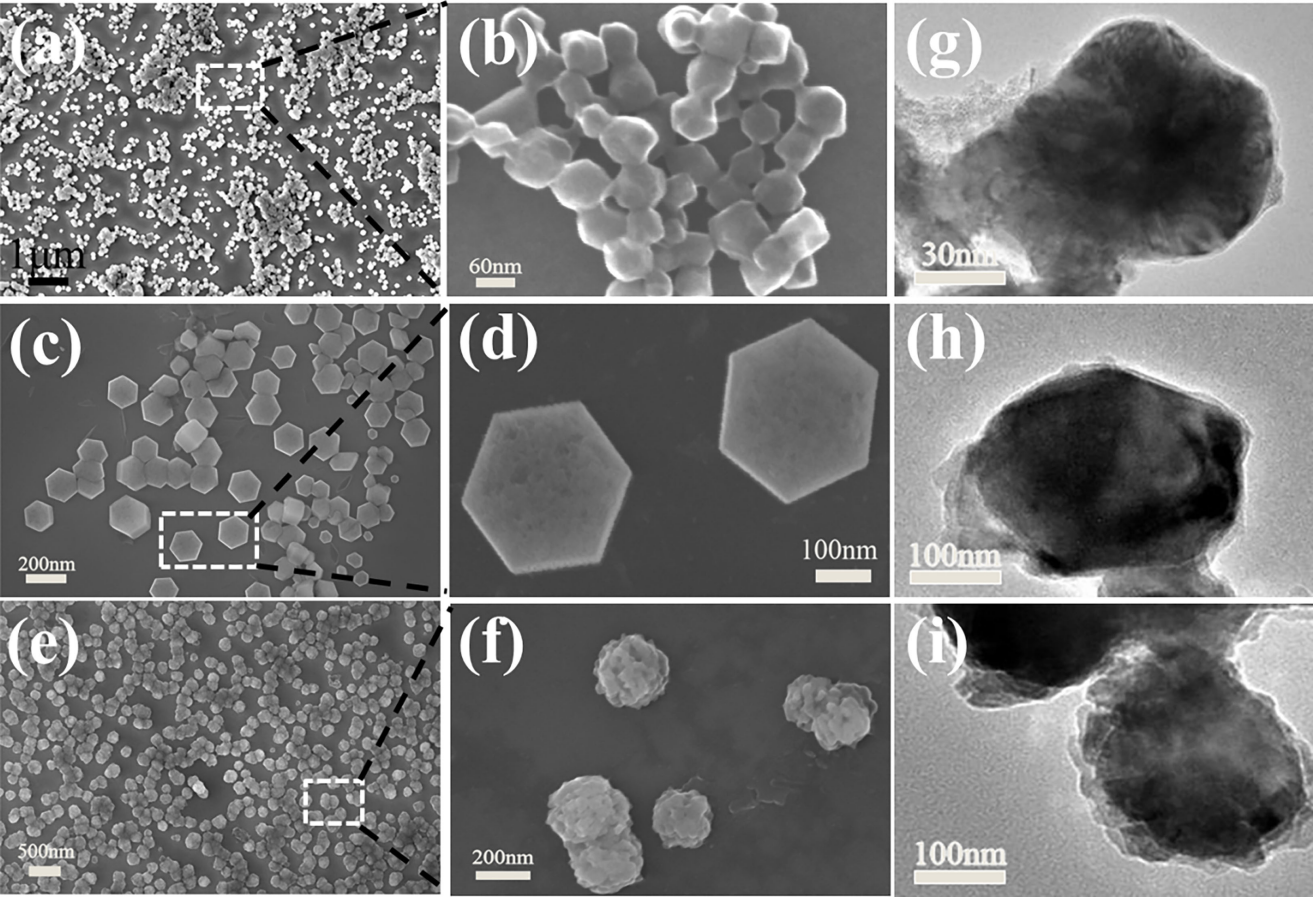
SEM of Zn-Co ZIF in **(A, B)**, ZD-C in **(C, D)**, ZD-CAR NPs in **(E, F)**. TEM of Zn-Co ZIF in **(G)** and ZD-C in **(H)**, and the TEM image of ZD-CAR in **(I)**.

ZD-CAR nanoparticles were characterized by SEM and TEM in **Figure 1**, and were characterized by Zeta potential, XRD, UV, FTIR, XPS and BET.

### 2.9 Photothermal performance testing

To evaluate the photothermal ability of ZD-CAR nanoparticles, the dispersions of aqueous solutions were irradiated under 808 nm NIR with different concentrations to observe the change in temperature with time; while their photostability was tested by repeatedly increasing and decreasing the temperature and withdrawing the laser after ten minutes of continuous illumination (52).

### 2.10 *In vitro* drug release

4 mg of ZD-CAR was dissolved in PBS at pH 7.4, 6.8 and 5.7. It was shaken in a constant temperature shaker at 37°C, and centrifuged at different time points. The same volume of supernatant was added to the same volume of PBS, and the wavelength of UV emission was measured at different times.

### 2.11 Tests for oxygen generation

Different concentrations of Zn-Co ZIF and ZD-CAR nanoparticles were added to an aqueous solution containing the same concentration of H_2_O_2_, and the O_2_ produced was tested using a JPB-607A dissolved oxygen instrument.

### 2.12 Cell culture

HepG_2_ cells were cultured in a medium supplemented with 10% fetal bovine serum (FBS) and 1% double antibodies (penicillin/streptomycin). Then, they were incubated in a 5% CO_2_ incubator at 37°C. The medium was changed every day. The cells were isolated with trypsin-EDTA, and they were passaged and seeded.

### 2.13 Cytotoxicity

The cytotoxicity of ZD-CAR *in vitro* was tested using HepG_2_ cells by CCK-8 reagent. A certain number of cells were inoculated in 96-well plates and incubated overnight in a 5% CO_2_ incubator at 37°C, after which the cells were washed with PBS. The same concentrations (25, 50, 100 and 200 μg/mL) of the different types of nanoparticles were placed in the medium and the cells were incubated. Three replicate wells were set up for each concentration. After 24 h incubation, the effect of the different concentrations of the drug on cell viability was measured using the CCK-8 kit. The absorbance of the well plates at 450 nm was measured using an enzyme marker, and cell viability was calculated. To test the effect of light duration on cell viability, PBS-treated cells and cells treated with 100 μg/mL ZD-CAR drug were used as controls and irradiated at different times. Then, the plates were stained using CCK-8 and the absorbance of the well plates at 450 nm was measured using the Tecan Sunrise. The following formula was used to calculate its cell viability:


(2) $$\text{Cell Viability (\%) = }\frac{{\text{OD}}_{\text{sample}}{\text{-OD}}_{\text{blank}}}{{\text{OD}}_{\text{control}}{\text{-OD}}_{\text{blank}}}\;\times \;100\%$$

where OD_control_ represents the absorbance of untreated cells and OD_blank_ means the culture without samples or cells and OD_sample_ indicates the cells with different treatments (38).

### 2.14 *In vitro* cellular hypoxia test

HepG_2_ cells were cultured under normoxic and hypoxic conditions respectively. The cells cultured under different conditions were stained with hypoxia-inducible factor (HIF-1α) and their fluorescence intensity was observed under a fluorescence microscope, after which the cells were incubated in normoxic and hypoxic environment using different materials, and cell viability was obtained after 24h of incubation using the CCK-8 kit to measure the absorbance of the well plates at 450nm using an enzyme marker.

### 2.15 *In vitro* treatment analysis

HepG_2_ cells were incubated overnight for wall attachment using confocal dishes. Then, they were treated with different types or concentrations of material and stained with different dyes. Subsequently, they were observed under confocal laser scanning microscopy (CLSM) or fluorescence microscopy.

### 2.16 Apoptosis assay

Apoptosis assay of hepatocellular carcinoma cells was performed using Annexin V-FITC/PI double staining apoptosis assay kit. HepG_2_ cells were inoculated into 6-hole plates at a density of 3×10^5^ per well and incubated overnight. The medium was aspirated and a fresh medium containing 200 μg/mL concentration of DOX, ZIF, and ZD-CAR, respectively was added. It was divided into six groups: addition of PBS with light only, addition of DOX, addition of ZIF, addition of ZD-CAR, and addition of ZD-CAR with light treatment. After it was incubated with drugs for 6 hours, the medium was discarded, and washed twice with PBS. Another fresh medium was added. The light only and ZD-CAR + light groups were exposed to 808 laser and power of 2 W·cm^-2^ for 5 minutes. Then, the cells were digested using EDTA-free trypsin. They were collected, washed, and centrifuged. The cells were treated with Annexin V-FITC/PI staining according to the manufacturer’s instructions. They were incubated for ten minutes at room temperature and protected from light. Then, they were analyzed using a flow cytometer on the machine.

### 2.17 *In vivo* treatment experiment

With the expected *in vitro* results, we established a HepG_2_ mouse model to determine the role of nanoparticles such as ZD-CAR in the treatment of tumours in mice *in vivo*. All experimental female mice (BALB/c-nu, 4-5 weeks old) were purchased from Guangdong Viton Lihua Laboratory Animal Technology Co., Ltd. Firstly, 1×10^6^ HepG_2_ cells were injected subcutaneously under the axilla of each mouse to establish the tumour model. Then, the mice were divided into 7 groups (5 mice per group) according to the materials, grouped as: PBS, Light, Zn-Co ZIF, ZD, DOX, ZD-CAR and ZD-CAR+L. The PBS group was treated with neutral PBS only as a control group, and the Light group was used as a pure light control group. For treatment, treatment was started when the tumour size reached about 120 mm^3^ and the treatment date was day 0. The same dose of drug was injected into the mice by tail vein injection for treatment and data such as the length and width of the tumour and its body weight were recorded for each mouse in each group every three days and the volume of the tumour (V) could be calculated by the formula. The formula is: V=0.5×(length)×(width)^2^.

## 3 Results and discussion

### 3.1 Characterization

As shown in **Figures 1A–F**, the SEM figures show that the size of the produced Zn-Co ZIF nanoparticles is around 80 nm; it has a clear granular shape. While, the size of the DOX-loaded and CS-coated nanoparticles is around 200 nm, showing a well-defined hexagonal shape with sharp angles. SEM graphics also showed that the embedding of DOX and the modification of CS did not change the morphology of the Zn-Co ZIF. The target product ZD-CAR is around 250 nm in size; the shape tends to be rounded and well dispersed, and a clear encapsulation of the outer layer can be seen. In **Figures 1G–I**, transmission electron microscopy reveals the apparently uniform morphology of the nanoparticle particles prepared at each step, and the attachment of the outer gold nanoparticles can be seen in **Figure 1I**.

**Figure 2:**
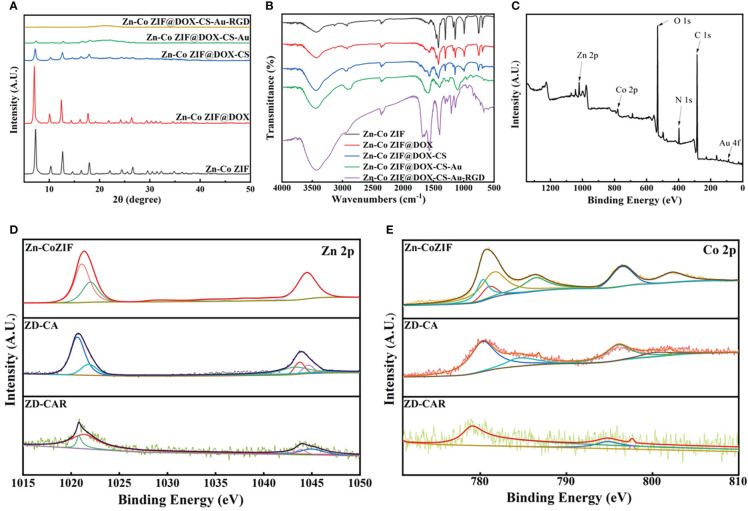
**(A)** XRD of Zn-Co ZIF, ZD, ZD-C, ZD-CA, ZD-CAR. **(B)** Infrared transmittance of Zn-Co ZIF, ZD, ZD-C, ZD-CA, ZD-CAR. **(C)** XPS of ZD-CAR. **(D)** XPS of Zn in every product and **(E)** XPS of Co in every product.

**Figure 3:**
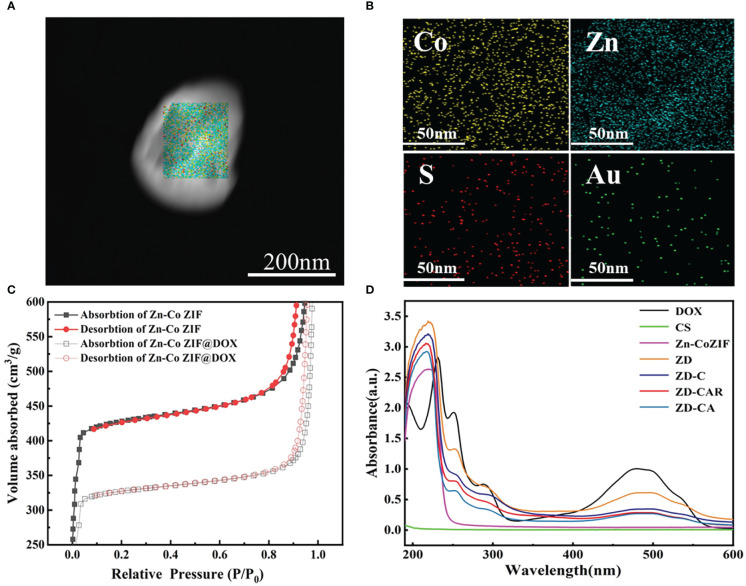
**(A)** STEM image. **(B)** TEM mappings of ZD-CAR. **(C)** BET of Zn-Co ZIF and ZD. **(D)** UV−vis spectroscopy of DOX, CS, Zn-Co ZIF, ZD, ZD-C, ZD-CA and ZD-CAR.

**Figure 4:**
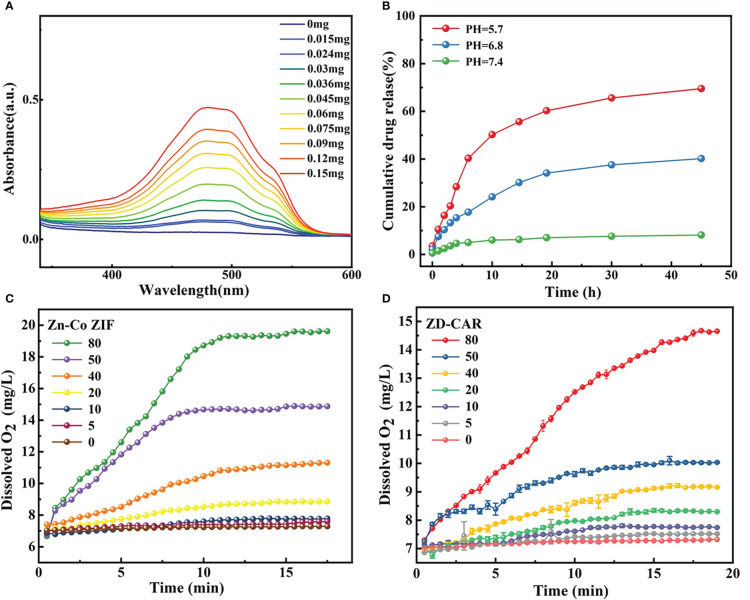
**(A)** UV−vis spectroscopy of ZD-CAR with different concentration. **(B)** Drug release process of ZD-CAR at different pHs at 37°C. **(C)** Oxygen production of Zn-Co ZIF and **(D)** Oxygen production of ZD-CAR (n=3).

**Figure 5:**
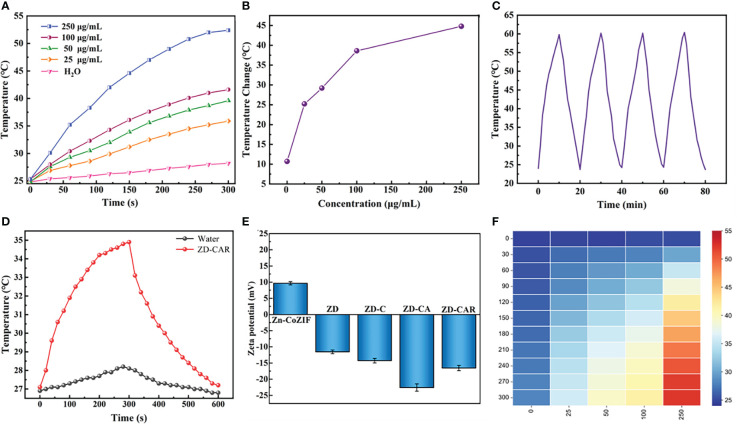
**(A)** Temperature changes of ZD-CAR with different concentrations under 808 nm laser irradiation (2W·cm^−2^). **(B)** Temperature changes of ZD-CAR with different concentrations under 808 nm laser irradiation for 10 min (2W·cm^−2^). **(C)** Recycling heating curves of ZD-CAR (1mg·mL^−1^) with laser (2W·cm^−2^). **(D)** Heating and cooling curves of water and ZD-CAR under 808 nm laser (2W·cm^−2^). **(E)** Zeta potential of Zn-Co ZIF, ZD, ZD-C, ZD-CA and ZD-CAR at 200μg/mL. **(F)** Heat map of ZD-CAR for temperature changes of ZD-CAR with different concentrations under 808nm laser irradiation (2W·cm^−2^).

As illustrated in **Figure 2A**, the XRD curves indicate that Zn-Co ZIF retains a sharp front after being loaded with DOX. This demonstrates the strong crystal structure of Zn-Co ZIF@DOX, and it also retains both characteristic peaks. This shows that the loading of DOX did not affect its crystal structure. The XRD of the chitosan-coated nanoparticles showed a reduction in the sharp edges, and after testing the XRD of the end-products, it was evident that the sharp edges disappeared. This shows that the attachment of RGD and Au nanoparticles did not affect the crystal structure. As shown in the FTIR transmission peak in **Figure 2B**, the overall peak position did not change, with the intensity of the group gradually increasing after each step of the material addition. In **Figure 2C**, X-ray photoelectron spectrometer (XPS) shows the characteristic peaks confirmed the presence of Au, N and S elements in the end product, further demonstrating that Au nanoparticles and disulfide bonds were successfully prepared. As shown in **Figures 2D, E**, XPS images indicate that Zn and Co elements decreased in peak position and content as the material was cladded and joined. This proves that the surface structure and elements changed. **Figures S1**, **S2** show the Raman signature peaks and Raman Spectroscopy of ZD-CAR. As shown in **Figures 3A, B**, the EDS of the material show that there were elements of Au, N and S in the ZD-CAR nanoparticles. This proves that the gold nanoparticles and sulfhydryl groups were successfully attached, and the material was successfully prepared. **Figure 3C** shows that the nitrogen adsorption isotherm results indicate that Zn-Co ZIF has a high specific surface area with more pores. This shows that Zn-Co ZIF has good drug loading potential and can be applied as a drug loading system. The lower specific surface area and smaller pore size volume of ZD compared to Zn-Co ZIF can help it to successfully encapsulate DOX. Testing the UV absorption of the products prepared in each step, it can be seen from **Figure 3D** that ZD-CAR contains the characteristic peaks of the drugs added in each step. This confirms the successful connection of each step and successful preparation of ZD-CAR nanoparticles. It is shown in **Figure S3** that Zn-Co ZIF loses its weight as the temperature rises. As shown in **Figure 4A**, testing the UV absorption peaks of different concentrations of nanoparticles shows that the absorption peaks increase significantly with increase in the mass added. The zeta potential test of the materials displayed in **Figure 5E** indicates that the materials have different potentials: Zn-Co ZIF had 9.65 ZD at -11.54 after loading with DOX and dropped to -14.27 after coating with chitosan; ZD-CA’s potential is -22.58 while ZD-CAR’s potential has -16.54.

### 3.2 *In vitro* drug release

The results in **Figure 4B** showed that the release of DOX in ZD-CAR was evaluated. The drug release rate was very low at pH=7.4; about 8% was released after 45 h. As the pH decreased, the ability of the ZD-CAR NPs to release DOX gradually increased. When the pH dropped to 5.7, the drug release rate increased to about 69.5%, and after 45 hours of oscillation, it was gradually levelled off after 30 h. It can be verified that in the weak acidic micro environment of the tumor there is an obvious released plateau.

### 3.3 Evaluation of the generation of oxygen

The dissolved oxygen of Zn-Co ZIF and ZD-CAR under the same conditions is tested as shown in **Figures 4C, D**. The same concentration of H_2_O_2_ was added, and the dissolved oxygen of both materials rises with increase in their concentration. With the addition of 80 μg/mL concentration and at about 15 min of reaction, the dissolved oxygen of bare Zn-Co ZIF could reach up to 20 mg/L; while that of ZD-CAR could get to 14 mg/L. Oxygen is produced due to the presence of Zn-Co ZIF (**Figure S4**). Oxygen is produced by Zn-Co ZIF due to the release of Co^2+^. It catalyzes the production of O_2_ from H_2_O_2_, and due to its porous structure, it can provide more active sites. These results indicate that the drug-carrying platform we prepared has good catalytic properties and potential to alleviate tumor hypoxia.

### 3.4 Photothermal effect test

In order to test the photothermal properties of ZD-CAR and determine whether it can be effectively used for photothermal therapy, the same volume of aqueous dispersions of ZD-CAR at different concentrations was irradiated with an 808 nm near infrared (NIR) laser and the solution temperature was monitored using thermocouple thermometer (OMEGA HH520). In **Figure 5A**, the temperature of the aqueous solution without ZD-CAR and at lower concentrations increased, but not significantly. As the laser irradiation time increased, the temperature of the aqueous dispersion increased significantly as the ZD-CAR concentration increased. The temperature of ZD-CAR could reach over 50 degrees Celsius when 250 μg/mL of ZD-CAR nanoparticle was irradiated for five minutes. At the same irradiation time, the temperature of the aqueous solution without ZD-CAR only increased by 10.7°C for ten minutes, but when 250 μg/mL of ZD-CAR nanoparticles was added, the temperature of the solution increased by 44.8°C (**Figure 5B**). More importantly, as shown in **Figure 5C**, after 4 consecutive cycles of irradiation with 808 nm laser, there was no significant fluctuation in the temperature change, proving that ZD-CAR has good photothermal stability. From **Figure 5D**, it can be seen that the temperature can return to room temperature, when the laser is removed after five minutes of continuous irradiation. According to **Figure 5D** and from equation (3), the photothermal conversion efficiency of the ZD-CAR can be calculated to be 47.1%, thus enabling photothermal conversion therapy. :


(3) $$\;\eta =\frac{hs\left(T_{max}-T_{surr}\right)-Q_{Dis}}{I\left(1-10^{-A_{808}}\right)}$$

It can be obviously explained in **Figure 5F** that the temperature changes at different concentrations and different times. In summary, it can be demonstrated that Zn-Co ZIF@DOX-CS-Au-RGD NPs have great photothermal conversion rates and photothermal stability for PTT treatment **(** 48, 53–55).

### 3.5 *In vitro* experiments

#### 3.5.1 Cytotoxicity

**Figure 6:**
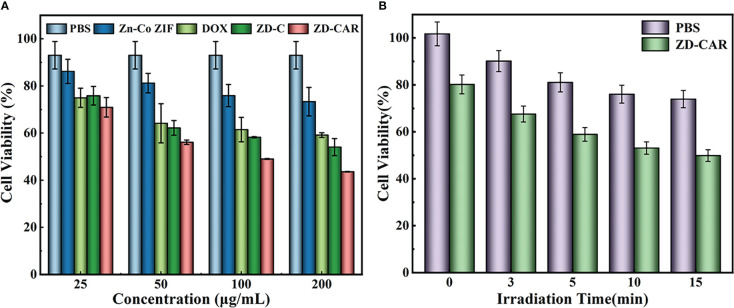
**(A)** Cell activity of different concentration with different treatment (incubate for 24 hours); **(B)** Cell activity of the cell incubated with different treatments under 808 NIR (incubate for 24 hours).

Based on the excellent catalytic properties and specific targeting ability of the measured ZD-CAR, we set up control groups with different concentrations and *in vitro* anti-cancer test using the cck8 kit. It was done to confirm the *in vitro* killing effect of our prepared nanoplatform on tumor cells, and to demonstrate its killing effect on tumor cells under light conditions to confirm its good photothermal effect. As the concentration of nanoparticles incorporated increased as shown in **Figure 6A**, the cellular activity gradually decreased. With the addition of 200μg/mL Zn-Co ZIF, the cellular activity was 73.36%, while the cell activity decreased to 43.59% after adding the same concentration of ZD-CAR. The IC50 of ZD-CAR was calculated to be 53.211μg/mL. A plot of the fitted curve for calculating the IC50 is shown in **Figure S5**. Compared to directly adding the chemotherapeutic drug DOX, the cytotoxicity of ZD-CAR nanoparticles against HepG_2_ was comparable, confirming their therapeutic efficacy. For the group with the same concentration of ZD-CAR, the cell viability of the light-treated cells showed significantly lower cell activity compared to the group without light-treated cells (**Figure 6B**), and the cell viability gradually decreased with increased light duration. The cell viability was about 49.86% after continuous exposure to light for 15min. This proves that the nanomaterial ZD-CAR has good photothermal sensitivity and can be applied in photothermal therapy.

#### 3.5.2 *In vitro* cellular hypoxia test

**Figure 7:**
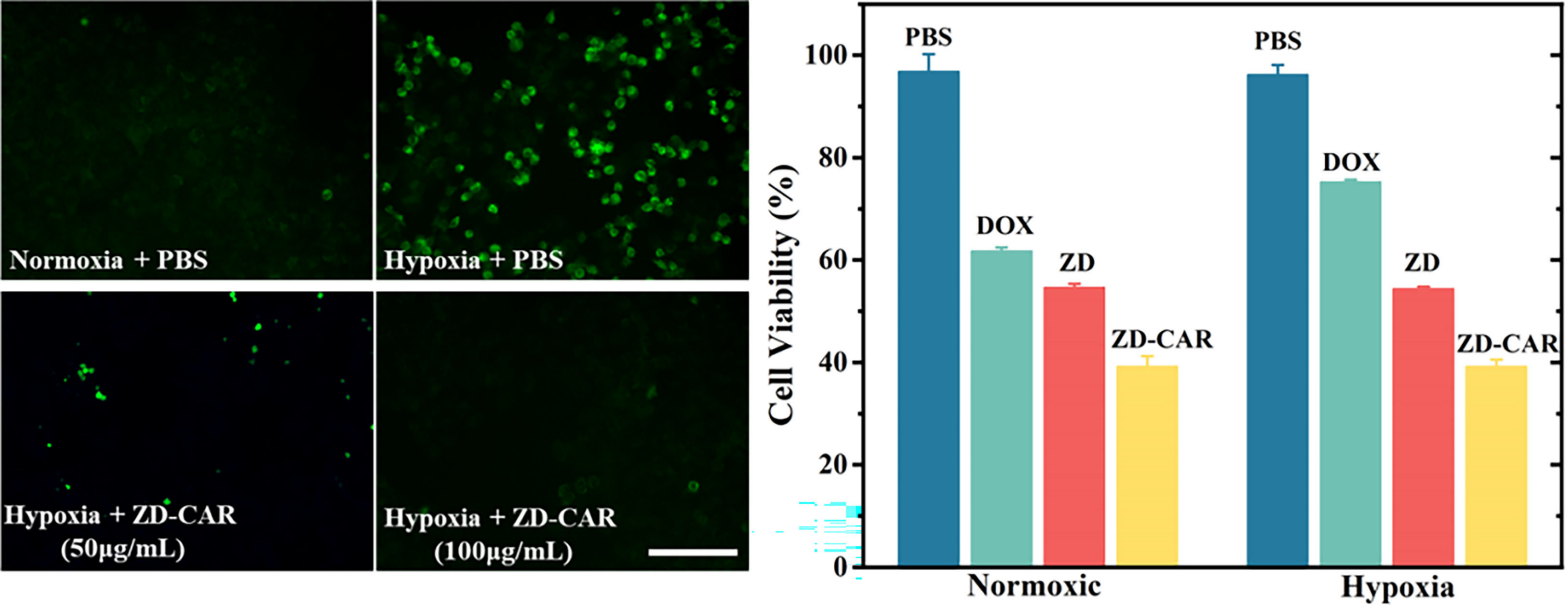
Fluorescence images of HIF-1α (green) in HepG_2_ cells incubated with ZD-CAR under normoxic or hypoxic conditions. Scale bar is 100 μm. (Left) and cell viability of different materials under normoxia or hypoxia (Right).

Hypoxia-inducible factor (HIF-1α) will upregulat under hypoxic conditions, so we used HIF-1α staining to detect the degree of hypoxia. As shown in **Figure 7** (Left), a weaker green fluorescence was observed when cells were cultured under normoxic conditions, while a stronger green fluorescence was observed in cells cultured under hypoxic conditions, consistent with the fact that hypoxia can induce HIF-1α accumulation. When cells were incubated with ZD-CAR under hypoxic conditions for 12 h, a concentration-dependent decrease in fluorescence intensity was observed, indicating that the nanoplatform could effectively alleviate intracellular hypoxia. As shown in **Figure 7** (Right), the cytotoxicity of ZD-CAR to HepG_2_ cells is independent of oxygen concentration, as ZD-CAR have the ability to alleviate hypoxia and exhibited the same high level of cytotoxicity to HepG_2_ cells under normoxic (21%) and hypoxic (1%) conditions. We know that DOX is more toxic in the presence of a certain amount of oxygen and has a better therapeutic effect. As shown in **Figure 7** (Right), in contrast, the viability of free DOX-treated cells was significantly higher under hypoxic conditions than under normoxic conditions. All these results confirm that the oxygen generated by ZD-CAR facilitates the efficacy of DOX and that ZD-CAR have superior cytotoxicity compared to the other groups, due to the effect of the combination of chemotherapy and targeted therapy.

#### 3.5.3 *In vitro* treatment analysis

**Figure 8:**
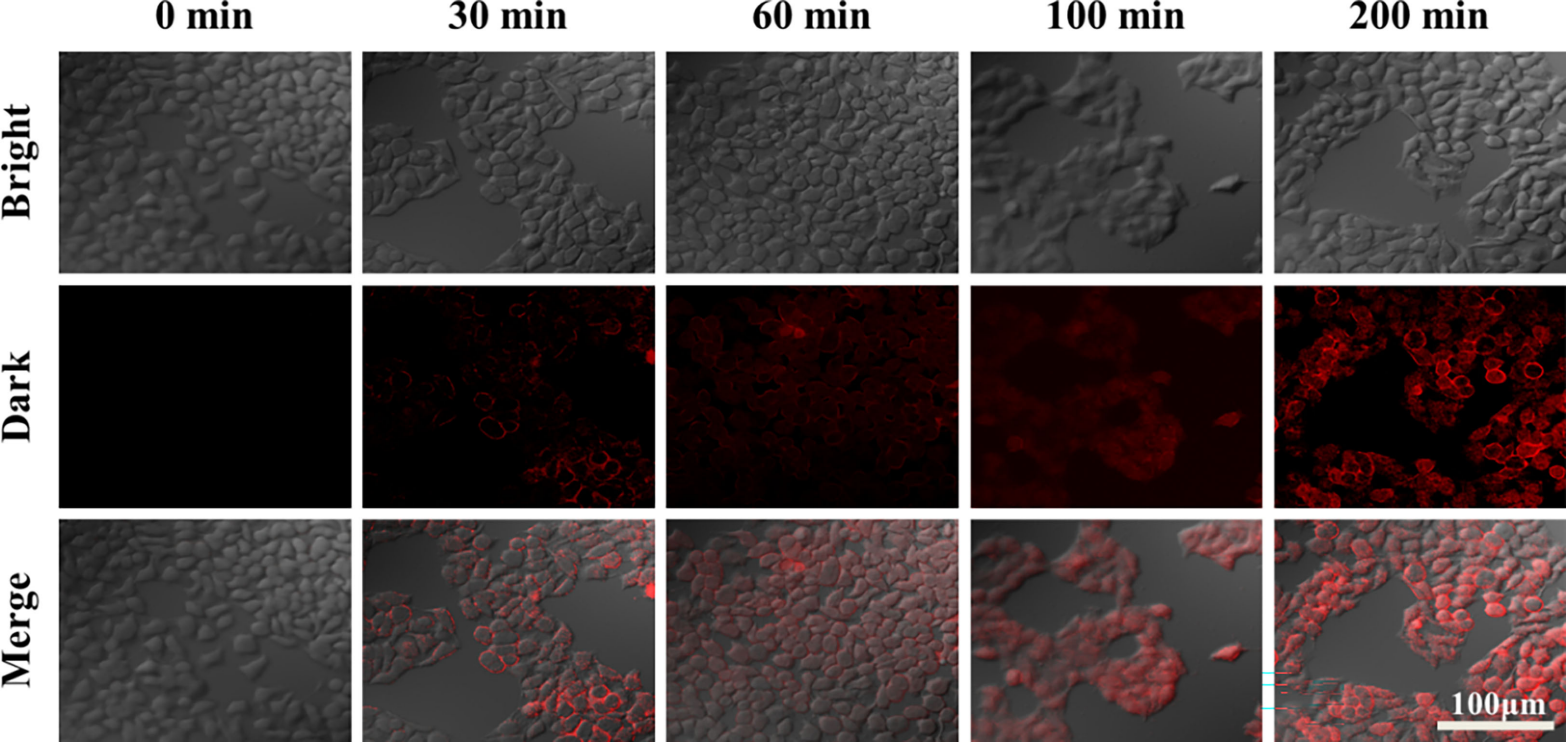
CLSM images of HepG_2_ cells after 200 μg/mL of ZD-CAR nanoparticles were incubated with different times (dyeing with PI).

As shown in **Figure 8**, HepG_2_ cells were treated with the same concentration of ZD-CAR nanomaterials at different incubation times. This was followed by PI staining and observation under confocal laser scanning microscopy (CLSM). It can be seen that a small number of cells started to die at around 30 min, and as the incubation time was extended, the cells reached a high mortality rate at 200 min. This shows that the material has good therapeutic efficacy.

**Figure 9:**
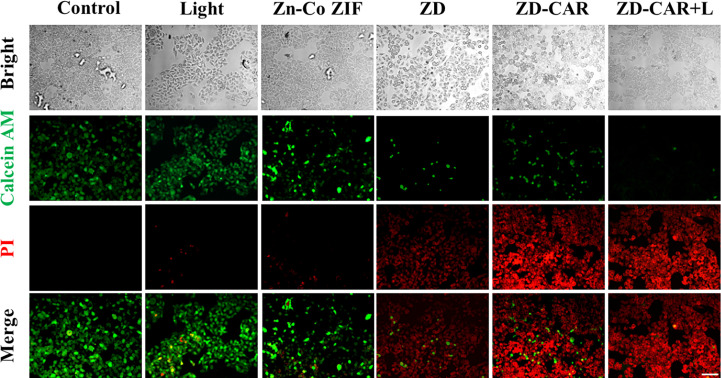
HepG_2_ cells treated with different materials were double stained with Calcein AM/PI (incubate for 24 hours). Scale bar is 100 μm.

As shown in **Figure 9**, cell mortality was negligible in the control and after 808 laser irradiation for only the Zn-Co ZIF treatment Cell mortality was significantly higher after the addition of DOX, which is the treatment effect produced by the chemotherapeutic drugs. More notably, compared to the ZD-CAR material only treatment, in the group treated with ZD-CAR and exposed to 808 light, almost all the cells died, which was the effect of the combined photothermal and chemotherapy treatment. This confirms the high efficiency of the combined treatment. In this experiment, staining was performed using Calcein AM/PI double staining.

**Figure 10:**
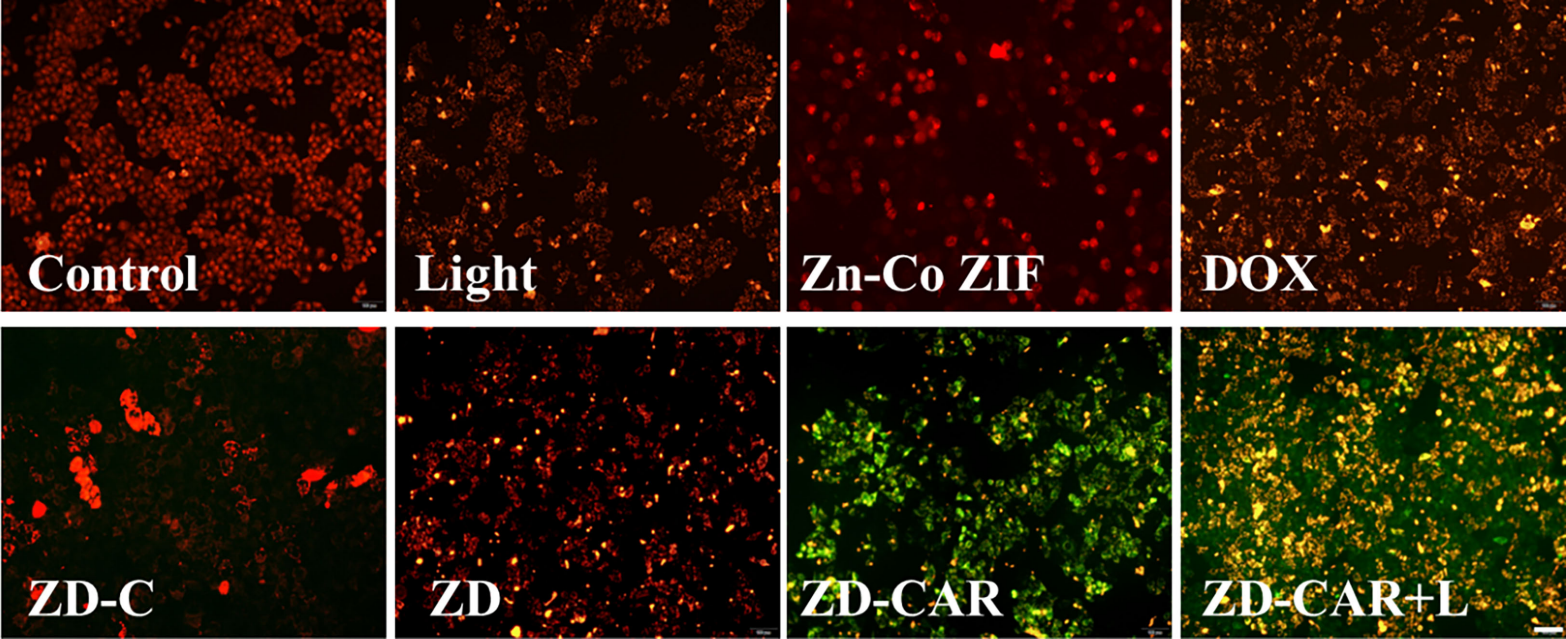
Jc-1 staining on HepG_2_ cells treated with different materials (incubate for 24 hours). Scale bar is 100 μm.

As shown in **Figure 10**, compared to the control group without any treatment, the cells showed a slight orange fluorescence after being exposed to light, and the red fluorescence of DOX-treated HepG_2_ cells diminished and began to show orange fluorescence. This demonstrates that the chemotherapy and photothermal treatment had an effect. However, after treating the cells simultaneously with ZD-CAR and 808 laser, almost all the cells showed green fluorescence. This shows that their mitochondrial function was severely affected by the combined treatment and could cause cell death with high efficiency. The ratio of fluorescence intensity was shown in **Figure S6**.

#### 3.5.4 Apoptosis assay (Flow cytometry)

**Figure 11:**
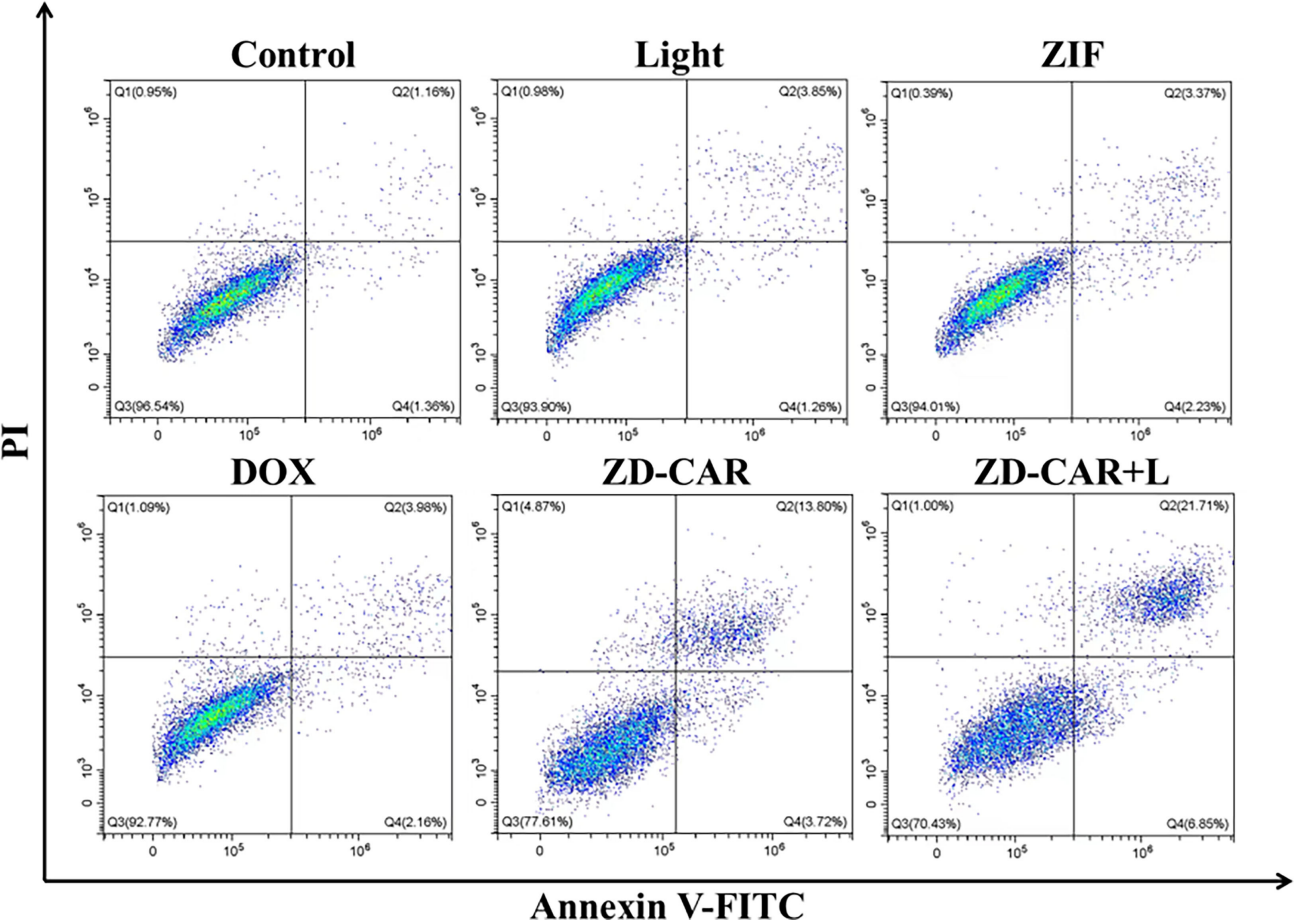
Flow cytometric after treating HepG_2_ cells in different ways using Annexin V-FITC staining (incubated for 24 hours on wall and 6 hours after addition of NPs).

As shown in **Figure 11**, the ZD-CAR+L incubation-treated group had a late apoptosis rate of up to 21.71% compared to the cells without treatment and the group treated with other drugs. The ZD-CAR-treated group had late apoptosis rate of 13.8%, but not the light-treated group. This confirms that ZD-CAR has a good photothermal conversion efficiency. The results are consistent with the cell viability measured by CCK8 and Calcein AM/PI double staining test. These results indicate that ZD-CAR has good biocompatibility and the combined treatment can provide high therapeutic efficiency for tumor cells.

#### 3.5.5 *In vivo* treatment experiment

**Figure 12:**
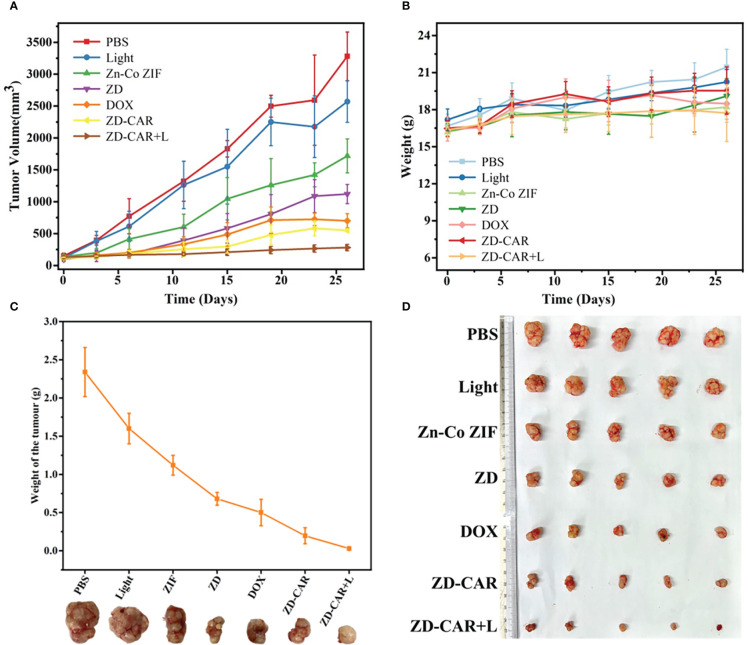
**(A)** The change curve of tumor volume in mice treated with different materials; **(B)** Body weight change curve of mice treated with different materials; **(C)** The change in weight of the tumour after removal of the tumour after treatment with different materials; **(D)** Tumours dissected from mice after execution.

PBS is a buffered solution that is commonly used as a solvent to disperse nanoparticles. The PBS group was used as a control group, the Light group to observe the effect of light alone on the growth of mice, and the Zn-Co ZIF group was set up to observe the effect of the metal-organic skeleton on the growth of mice. After 26 days of treatment and recording the data, **Figure 12** was obtained. As shown in **Figure 12A**, after adding the same concentration of different drugs for treatment, the growth trend of mice volume was significantly different, the mice in the PBS and Light groups grew rapidly, and it can be known that light alone didn’t affect the growth of mice. Compared with the other groups, the ZD-CAR+L group had the most obvious inhibitory effect on tumour volume increase, proving that it had the best therapeutic effect. As shown in **Figure 12B**, the change in body weight of each mouse was also recorded throughout the treatment, with an overall trend of growth, but no significant difference between the groups, indicating that the treatment material we designed has good biocompatibility and did not affect the basic physiological growth of the mice, with no significant side effects. After 26 days of treatment, the mice were all executed for further observation. As shown in **Figure 12D**, the tumours in the mice treated with ZD-CAR+L had the smallest volume of all groups, demonstrating the good tumour treatment effect of the material. Each tumour was weighed to obtain **Figure 12C**, and as can be seen from the curves in the figure, the mean tumour weight was different in each group and the ZD-CAR+L treated group had the smallest tumour weight, which is consistent with that shown in **Figure 12D**.

**Figure 13:**
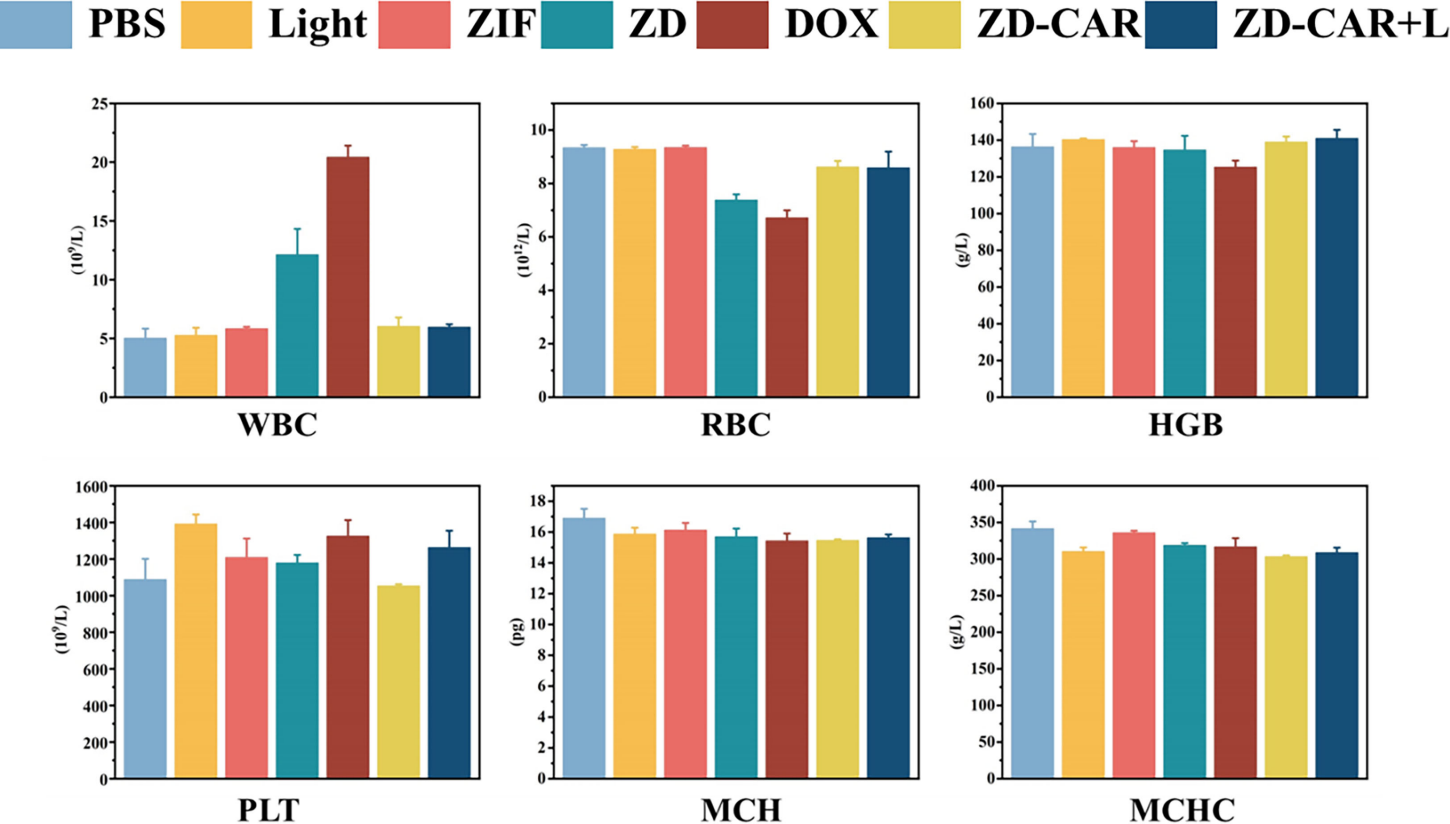
Routine blood tests of mice treated with different materials.

Blood is an important component in maintaining the vital signs of animals. After execution of the mice, blood was obtained from the mice by means of eye blood sampling and routine blood tests were performed to obtain **Figure 13**. WBC is the white blood cell count and as shown in the figure, there was no significant difference between the Light, Zn-Co ZIF, ZD-CAR and ZD-CAR+L groups and the PBS group, however, the ZD and DOX groups had several times more white blood cells compared to the other groups. RBC is the red blood cell count, as shown in the figure, which is consistent with the white blood cell results, only the ZD and DOX groups had a decrease in red blood cell count. PLT is the platelet count and the graph shows no significant difference between the groups. MCH represents mean erythrocyte haemoglobin content and the normal range is 15.8-19 (pg) and the test results show no significant difference between the groups. MCHC represents mean erythrocyte haemoglobin concentration and the normal range is 302-353 (g/L) and from the data obtained it can be seen that the data for the groups under different material treatments. There were no significant differences in the data obtained. Based on the results of all blood tests, it is clear that the ZD-CAR nanoparticles we prepared are biocompatible and have no effect on the normal survival of mice, whereas the diffusion of free DOX and the group ZD loaded with DOX can have toxic side effects on mice due to the lack of outer coating, resulting in an increase in the number of white blood cells and a decrease in the number of red blood cells, which verifies the success of the ZD-CAR nanoparticle This validates the success of the ZD-CAR nanoparticle preparation, which is in line with the original intention of producing therapeutic but not toxic side effects.

**Figure 14:**
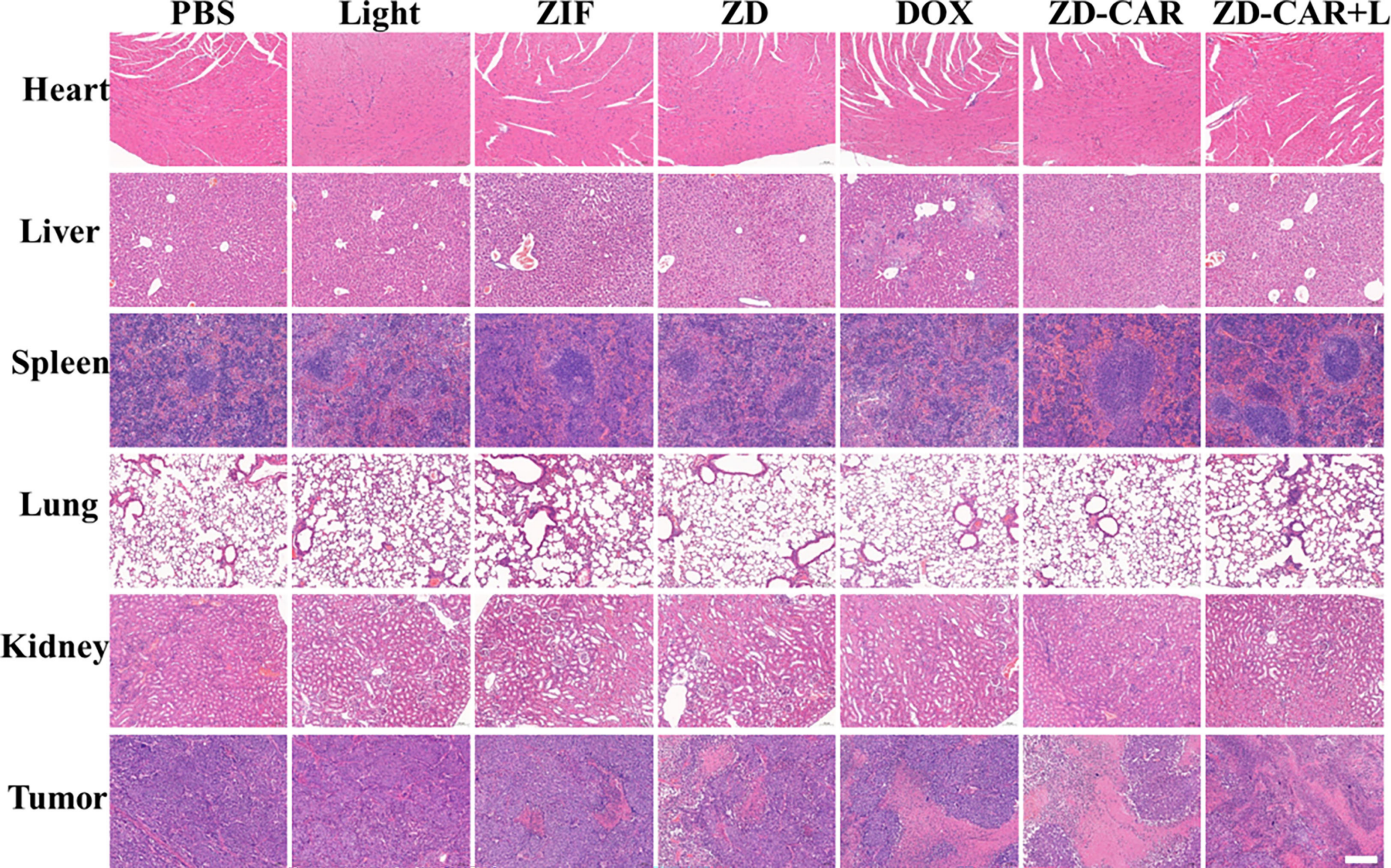
Fluorescence microscopy images of H&E tumor tissues after various treatments. (Scale bar is 100μm).

In order to investigate whether our Zn-Co ZIF@DOX-CS-Au-RGD has good biosafety, one mouse from each group was randomly selected for execution at the end of treatment, and the heart, liver, spleen, lungs and kidneys were sectioned and fixed with tissue fixative Paraformaldehyde (4%) was used for fixation, followed by histological analysis. The results of the experiments are shown in **Figure 14** below. From the figures, it can be seen that the final prepared ZD-CAR did not differ significantly from the control group in all tissues *in vivo*, indicating that it has good biocompatibility and biosafety.

**Figure 15:**
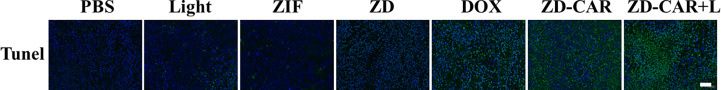
Fluorescence microscopy images of Tunnel-stained (green) tumor tissues after various treatments. (Scale bar is 50μm).

Terminal deoxynucleotidyl transferase-mediated dUTP nickel-end labeling (TUNEL) staining further confirmed that the ZD-CAR and ZD-CAR+L groups had more apoptotic cell regions than the control group (**Figure 15**), indicating that this tumour vascular targeting strategy enhanced tumour growth inhibition *in vivo*.

## 4 Conclusions

In summary, a MOF ZD-CAR material containing both Co^2+^ and Zn^2+^ was successfully prepared in this study. Using the pH-sensitive property of easy cleavage under acidic conditions, it can be smoothly cleaved to release Co^2+^ and DOX under the tumor microenvironment. Co^2+^ can catalyze H_2_O_2_ to produce O_2_ to alleviate the tumor hypoxic environment, while the DOX released can be used for chemotherapy. In the meantime, the attachment of gold nanoparticles can confer good photothermal sensitivity for photothermal therapy, and secondly, the SH-RGD attached to the surface of the nanoparticles has good tumor targeting, so it cannot harm healthy tissues. After the material was prepared, its various components were characterized and various tests were done for its performance. The *in vitro* material tests and experiments such as cytotoxicity and confocal confirm the good therapeutic effect of the ZD-CAR nano-drug delivery platform, which provides a new idea for the combined chemotherapy and photot hermal treatment.

## Data availability statement

The original contributions presented in the study are included in the article/**Supplementary Material**. Further inquiries can be directed to the corresponding authors.

## Author contributions

CY: Investigation, Data Curation, Writing – Original Draft. ST: Validation, Formal analysis, Writing – Review & Editing. LG: Methodology, Supervision. GA: Resources, Writing – Review & Editing. HZ: Data Curation, Writing – Review & Editing. JH: Writing – Review & Editing. LJ: Investigation. ZB: Formal analysis. YZ: Funding acquisition, Project administration. NW: Methodology, Conceptualization, Supervision. All authors contributed to the article and approved the submitted version.
