# Supplementary material for: Zn-Co metal organic frameworks coated with chitosand and Au nanoparticles for chemo-photothermal-targeted combination therapy of liver cancer
Source: Front Oncol. 2023 Apr 19;13:1110909. doi: 10.3389/fonc.2023.1110909 (PMC10154549; doi:10.3389/fonc.2023.1110909)
Supplement: Supplementary file 1 [file Table_1.docx]

**Supporting Information**

**Materials**

# 2-Methylimidazole (C_4_H_6_N_2_, 2-MIM), Doxorubidn hydrochloride (DOX) , chitosan (CS) and mercaptopropionic acid (MPA) , were purchased from Shanghai Macklin Biochemical Technology Co., LTD, zinc nitrate hexahydrate (Zn(NO3)_2_•6H_2_O), chloroauric acid, were purchased from Sinopharm Chemical Reagent Co., LTD, cobaltous nitrate hexahydrate (Co(NO_3_)_2_-6H_2_O), and methanol were purchased from Guangdong Guanghua Sci-Tech Co., LTD. 1-Ethyl-(3-dimethylaminopropyl) carbodiimide hydrochloride (EDC) , N-Hydroxysuccinimides (NHS) were purchased from Shanghai Aladdin Biochemical Technology Co., LTD. SH-RGD was purchased from Xi'an Ruixi Biotechnology Co., LTD, sodium borohydride was purchased from Tianjin Kermel Chemical Reagent Co., LTD, hydrogen peroxide (H_2_O_2_,30%) was purchased from Chengdu Jinshan Chemical Reagent Co., LTD, Propidium iodide (PI, for dead cell staining) was purchased from Beijing Lanjeke Technology Co., LTD, Calcein-AM, (for live cell staining),was purchased from and dimethyl sulfoxide (DMSO) were purchased from Beijing Solar bio Co., LTD. DMEM, fetal bovine serum, trypsin, Penicillin/Streptomycin Dual Antibody were purchased from Nanjing Wisent Co., LTD.

**Instrument type**

Scanning electron microscopy (SEM, Sigma-300, Zeiss, UK) at 5 kV and transmission electron microscopy (TEM; FEI TalosF200x, USA) at 200 kV was used to observe the surface morphology of the samples. X-ray diffraction (XRD, Rigaku D/MAX 2500 V, Japan) with Cu Kα radiation (100 mA, 40 kV) at a scanning rate of 5° min−1 was used to characterize and analyze the crystal structure of the samples. The elemental composition and valence states of the samples were analyzed by X-ray photoelectron spectroscopy (XPS, Axis Ultra DLD, England). The defect density and degree of graphitization of the samples were analyzed using a laser Raman spectrometer (inVia Reflex, UK) .The adsorption and desorption isotherms of nitrogen were obtained by using an automatic specific surface area analyzer (TriStar II 3020, USA), and the Brunauer–Emmett–Teller (BET) was used to obtain the corresponding specific surface area.


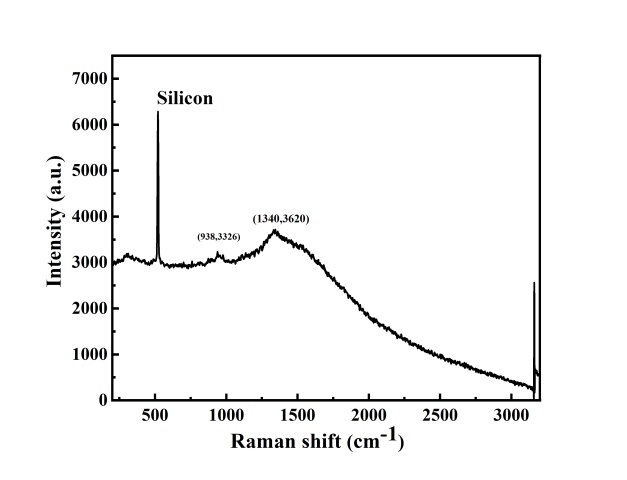


Figure S1: Raman of ZD-CAR.


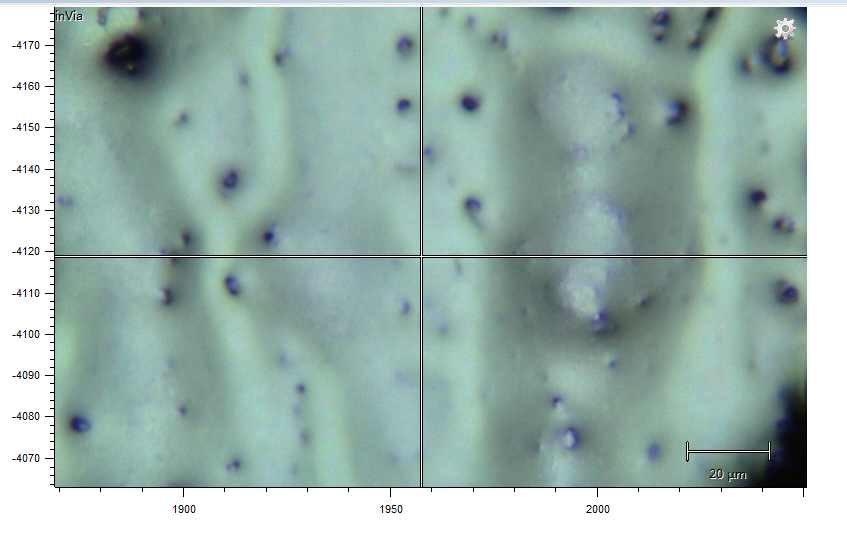
 Figure S2: Raman Spectroscopy of ZD-CAR.


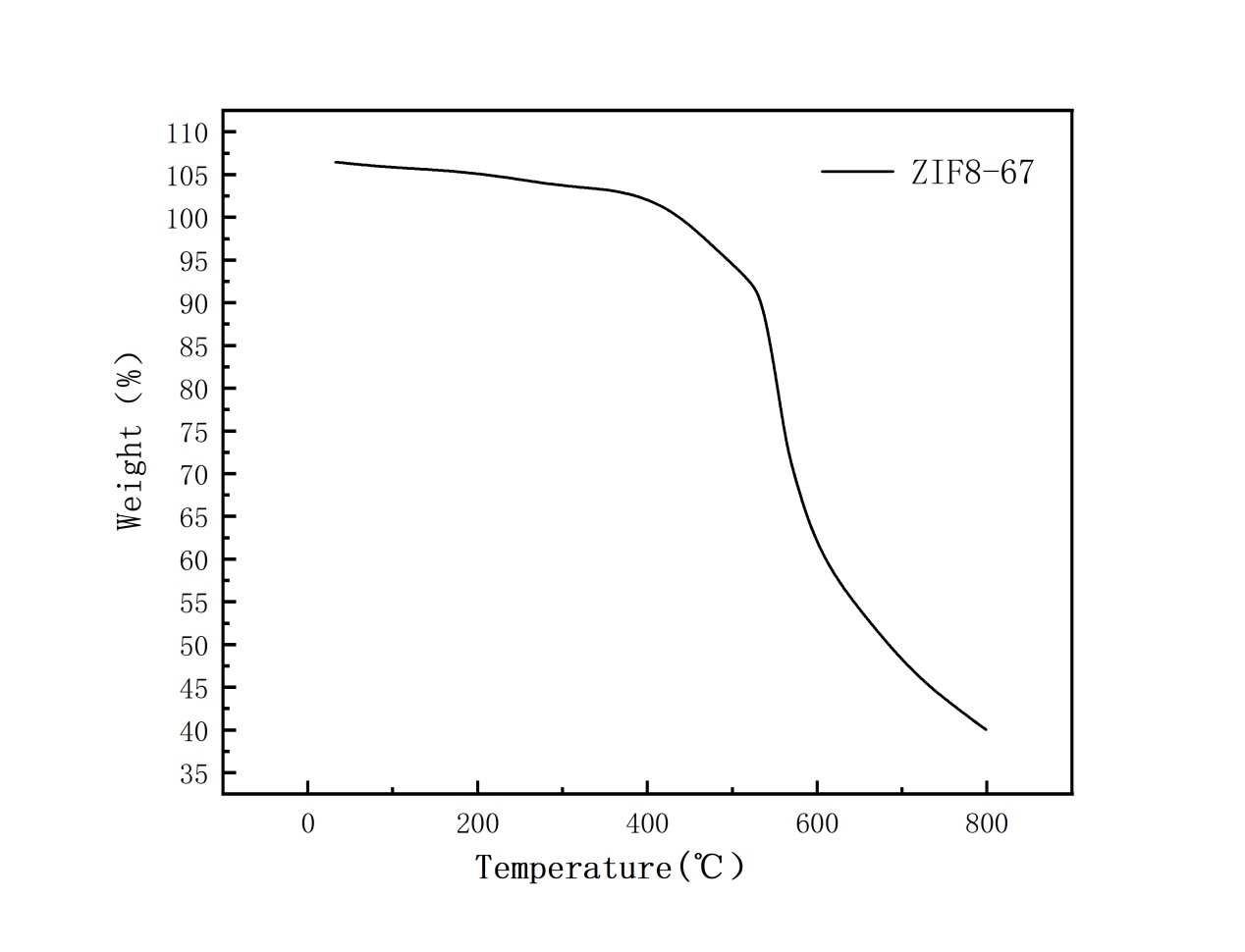


Figure S3: Thermo gravimetry of Zn-Co ZIF.


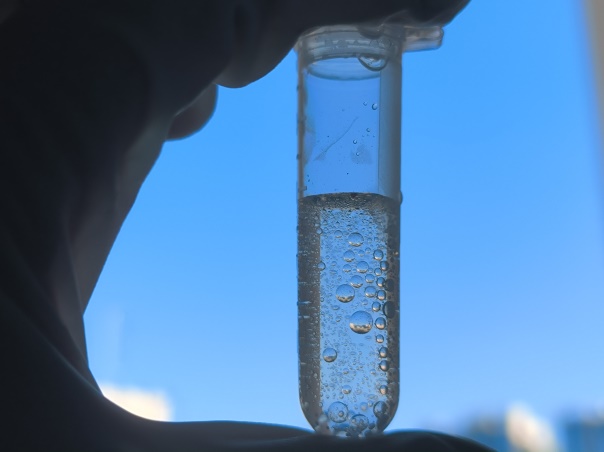

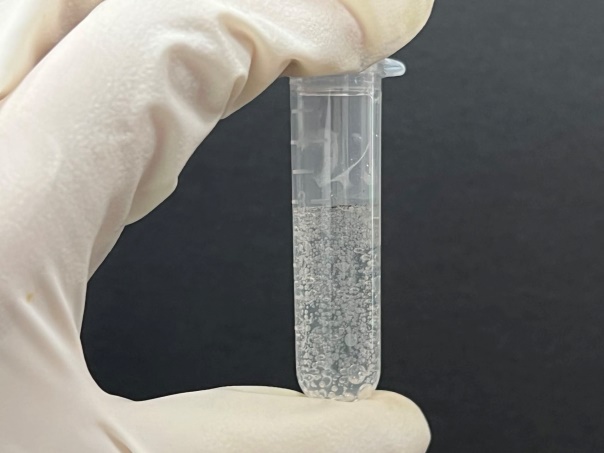


Figure S4: Pictures of dissolved oxygen of ZD-CAR (Left) and Zn-Co ZIF (Right).


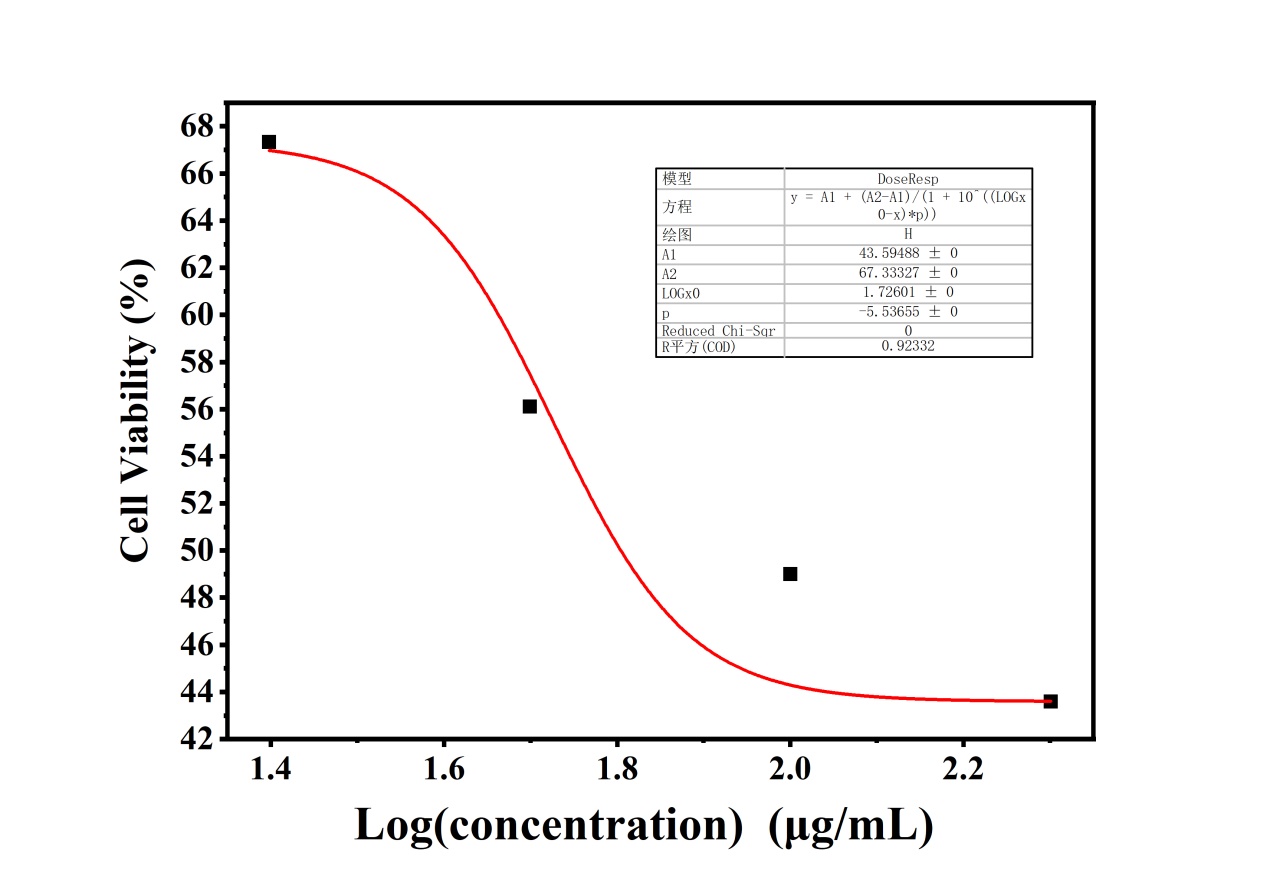


Figure S5: Fitted graph for calculating IC50.


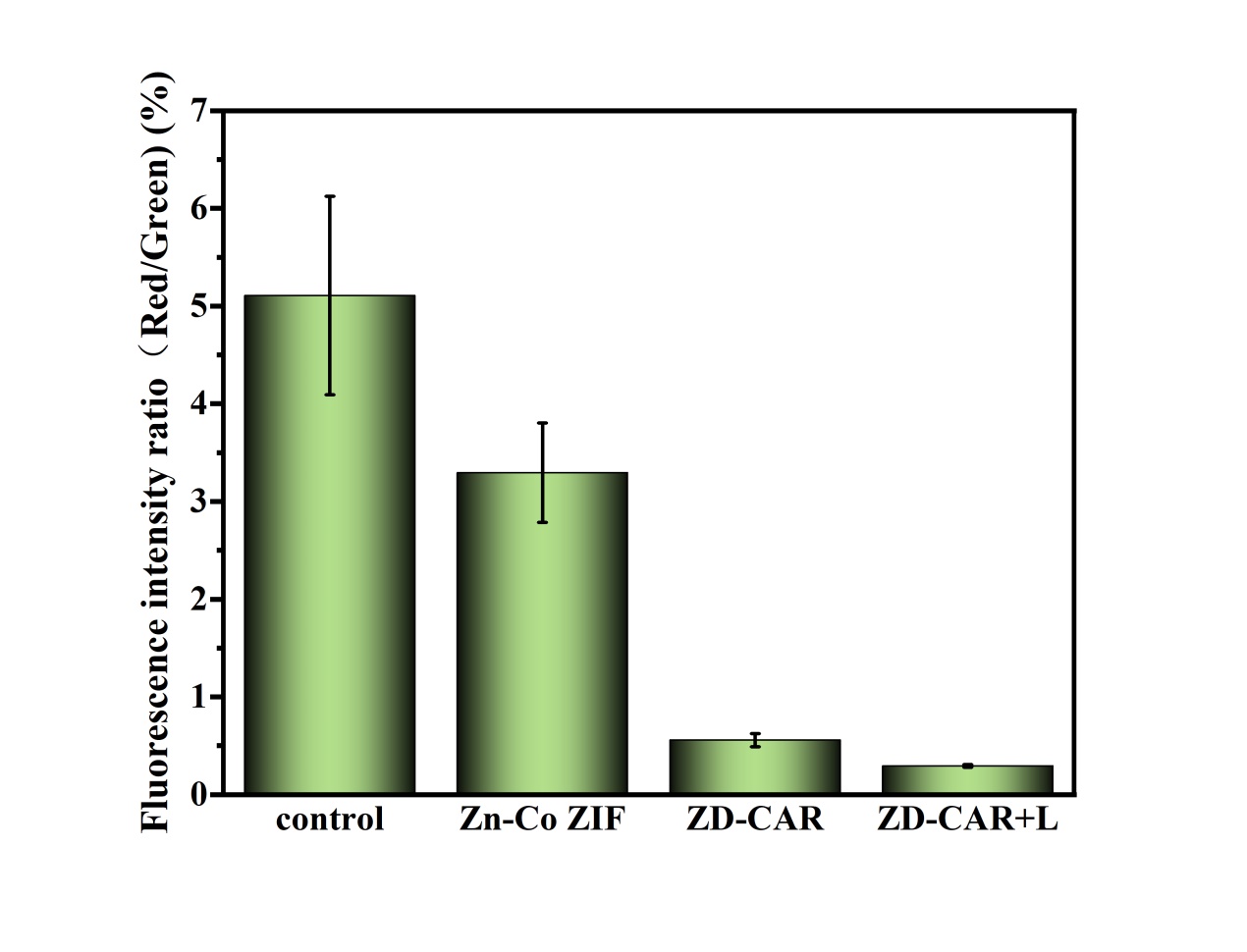


Figure S6：Ratio of fluorescence intensity.(Red/Green) (n=3)
